# Association between vitamin C intake and thyroid function among U.S. adults: a population-based study

**DOI:** 10.3389/fendo.2024.1462251

**Published:** 2024-11-07

**Authors:** Jie Wu, Chuyu Jia, Qiang Wang, Xin Li

**Affiliations:** ^1^ Department of thyroid surgery, Shanxi Provincial People’s Hospital, Taiyuan, Shanxi, China; ^2^ Department of physical examination center, Shanxi Provincial People’s Hospital, Taiyuan, Shanxi, China

**Keywords:** vitamin C, thyroid function, alcohol, NHANES, cross-sectional analysis

## Abstract

**Background:**

Although some evidence suggests a role for vitamin C intake in thyroid diseases, the complex interplay between vitamin C intake and thyroid function remains incompletely understood. The objective of this study was to explore the relationship between vitamin C intake and serum thyroid function in the United States adults.

**Methods:**

A total of 5,878 participants from the National Health and Nutrition Examination Survey (NHANES) between 2007 and 2012 were included in this study. Weighted multivariate linear regression models, subgroup analyses, and interaction terms were used to assess the association between vitamin C intake, evaluated as a continuous and categorical variable, and thyroid function. Additionally, restricted cubic spline (RCS) regression was employed to assess any nonlinear relationship that may exist between vitamin C intake and thyroid function.

**Results:**

After adjusting for covariates, our research found a significant inverse correlation between vitamin C intake and total thyroxine (TT4) (β= -0.182, *P=* 0.006). Using subgroup analyses, the association was more pronounced among subjects with lower alcohol consumption(β= -0.151, *P=*0.013, *P* for interaction = 0.043). In RCS regression, the correlation between vitamin C and TT4 exhibited a distinct reversed L-shaped curve pattern in total participants (*P* for nonlinear = 0.005) and male adults (*P* for nonlinear = 0.014). Additionally, we found an inverted U-shaped curve pattern in the relationship between vitamin C intake and FT4 (*P* for nonlinear = 0.029), while an U-shaped curve relationship was observed between vitamin C consumption and the FT3/FT4 ratio (*P* for nonlinear = 0.026).

**Conclusion:**

The findings of our study have illustrated a notable correlation between vitamin C intake and thyroid function. A high level of vitamin C intake is associated with a decreased in TT4 levels among American adults, and the association was more pronounced among subjects with lower alcohol consumption. Furthermore, our analysis revealed a nonlinear correlation between the intake of vitamin C and the levels of TT4, FT4, and FT3/FT4 ratio. Our findings support the rationale for making food-based dietary recommendations and maybe provide guidance for diet guidelines with thyroid dysfunction to a certain extent in the future.

## 1 Introduction

Thyroid, a crucial endocrine gland, regulates various functions in the body, such as metabolism, growth, and development through the secretion of thyroid hormones (1). The incidence of thyroid dysfunction varies by factors including age (2), sex (3), diet (4) geography (5), and genetics (6). For example, women are more likely to have thyroid problems at certain ages, such as puberty, pregnancy, and menopause, due to fluctuating hormone levels. In addition, factors such as environmental pollution (7), poor living habits (8), and trace elements (9, 10) may also increase the risk of thyroid disease. According to epidemiological data, the incidence of thyroid-related diseases has increased and become a health concern of global worldwide. A nationally representative cross-sectional study has revealed that the overall prevalence of thyroid dysfunction among adults stands at 15.17%. Specifically, subclinical hypothyroidism accounts for 12.93% of the cases, overt hypothyroidism comprises about 1.02%, subclinical hyperthyroidism constitutes 0.44%, and overt hyperthyroidism represents 0.78% of the total prevalence (11).

Vitamin C is an essential nutrient that plays a pivotal role in various biochemical processes within the body, including enzymatic activation, antioxidant reactions, and the maintenance of immune functions (12, 13). Unlike other animals, humans lack the ability to synthesize vitamin C internally. Therefore, we must obtain sufficient amounts of vitamin C from food or supplements to maintain good health. In recent years, there has been increasing interest in exploring the potential link between vitamin C and thyroid function and thyroid disorders. Several studies have shown that vitamin C kills thyroid cancer cells by inhibiting signaling pathways via distinct mechanisms (14–16). In addition, lack of vitamin C can make thyroid tissue more susceptible to oxidative stress, which can increase the risk of goiter development. And appropriate supplementation of vitamin C can effectively reduce this oxidative stress and lower the incidence of goiter (17).

The majority of existing studies have concentrated on the effects of vitamin C on specific thyroid disorders or its interaction with thyroid medications, rather than exploring its broader impact on thyroid function and hormone levels. Therefore, the objective of this study was to investigate the correlation between vitamin C intake and serum thyroid function among adults in the United States, employing data obtained from the National Health and Nutrition Examination Survey (NHANES).

## 2 Methods

### 2.1 Study population

NHANES is a program of studies designed to assess the health and nutritional status of adults and children in the United States. Through a combination of interviews and physical examinations, NHANES provides insights into the prevalence of major diseases and conditions, dietary and nutritional patterns, and health-related behaviors in the United States. Conducted by the National Center for Health Statistics (NCHS) at the Centers for Disease Control and Prevention (CDC), all participants were required to sign the informed consent document prior to the commencement of the investigation.

**Figure 1:**
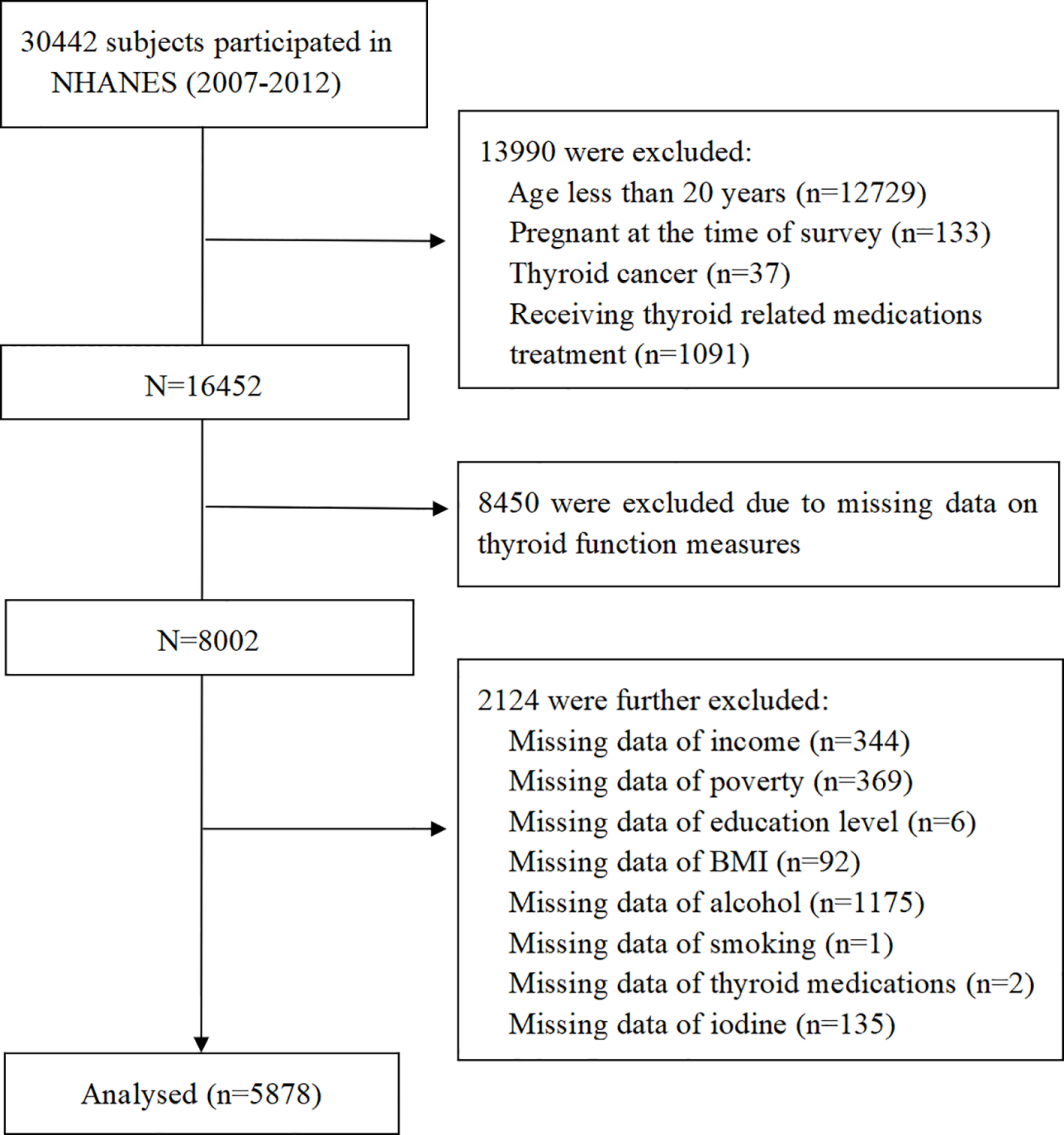
Flowchart of study population.

For the current study, a total of 30,442 participants were identified and selected from the NHANES cycles between 2007 and 2012. Exclusion criteria were: (1) age of participants was < 20 years or participants were currently pregnant; (2) participants with thyroid cancer or receiving thyroid-related medication treatment; (3) participants without thyroid measurement data; (4) participants without vitamin C intake data; (5) participants with missing covariate values. Finally, 5,878 participants were incorporated into our analysis (**Figure 1**).

### 2.2 Serum thyroid measure

The thyroid panel comprises a series of tests intended for evaluating thyroid gland activity. This panel includes determinations of thyroid stimulating hormone (TSH), total thyroxine (TT4), free thyroxine (FT4), total triiodothyronine (TT3), free triiodothyronine (FT3), thyroglobulin antibodies (TgAb), and thyroid peroxidase antibodies (TPOAb). Thyroid blood specimens were processed, stored and shipped to University of Washington, Seattle, WA. Detailed specimen collection and processing instructions are discussed in the NHANES Laboratory/Medical Technologists Procedures Manual (LPM).

### 2.3 Dietary vitamin C intake assessment

Dietary vitamin C intake was derived from the Food and Nutrient Database for Dietary Studies (FNDDS), which is utilized to code and analyze dietary intakes for the What We Eat In America (WWEIA), NHANES. Dietary vitamin C intake was calculated based on the average of two 24-hour dietary recalls. The initially collected recall was obtained in-person at the Mobile Examination Center (MEC), while the second interview was conducted over the phone after an interval of 3 to 10 days. In this study, the total intake of vitamin C includes a variety of foods and vitamin C supplements. Furthermore, due to the non-normal distribution characteristics of the vitamin C intake data, we implemented logarithmic transformations in order to obtain normal distributions of the data (named lnVc), which was subsequently confirmed by the conduct of Shapiro-Wilk tests. The lnVc were categorized into quartiles as follows: quartile 1 (0-1.508); quartile 2 (1.509-1.828); quartile 3 (1.829-2.074), and quartile 4 (2.075-3.037).

### 2.4 Covariates

According to the previous studies, we considered as confounding factors, information on demographics, socioeconomic status, diet, and health conditions, such as age, gender, race/ethnicity, education level, annual family income, alcohol consumption, poverty to income ratio (PIR), body mass index (BMI), urinary iodine concentration (UIC) and smoking (18). Considering the effects of other trace elements on the thyroid function, we also included dietary vitamin A(Va) (19), vitamin D(Vd) (20), vitamin E(Ve) (21), zinc(Zn) (22) and selenium(Se) (23) intake in this study.

Age was grouped into three groups: 20-40, 41-60 and more than 60 years. Education level was classified into three categories: less than high school, high school graduate, more than high school. Race was grouped into non-Hispanic white, non-Hispanic black, Mexican American, and others. Annual family income was divided into three groups (< 25,000, 25,000 to 74,999 and ≥75,000$). Poverty was defined as PIR less than 1.0, and PIR is further classified into four categories(<1.0, 1.0-1.9, 2.0-3.9, ≥4.0). BMI was categories as <25.0, 25.0–29.9, and ≥30 kg/m^2^. Alcohol consumption was defined as the average daily intake of any type of alcoholic beverage over the past year. A previous study on thyroid function have classified alcohol consumption into three categories of ≤50 g/day, 51 to 100 g/day, and > 100 g/day, and we adopt this classification method as well (24). UIC to determine iodine conditions in participants, which was classified as <100, 100-299 and ≥300 ug/L (25). Smoking habits were categorized as never, former, or now.

### 2.5 Statistical analysis

All statistical analyses were performed using the R package. Continuous variables were expressed in terms of the mean (standard deviation, SD), while categorical variables were summarized as frequency and percentage. With regard to the baseline characteristics, categorical variables were analyzed using the chi-square test for comparison, and continuous variables were analyzed using the one-way analysis of variance.

Weighted multivariate linear regression models were implemented to analyze the potential correlation between lnVc consumption and thyroid function indices. Model 1 was unadjusted for any covariates, whereas Model 2 underwent adjustments for a comprehensive array of factors including age, gender, education level, race/ethnicity, PIR, BMI, UIC, alcohol consumption, annual family income and smoking habits. Based on Model 2, Model 3 further adjusted for additional confounders, including Va, Vd, Ve, Zn and Se. Subgroup analysis, interaction tests and restricted cubic spline (RCS) regression were employed to evaluate the nonlinear association between lnVc and thyroid function. P value < 0.05 was considered statistically significant.

## 3 Results

### 3.1 Baseline participant characteristics

This study included 5,878 US participants aged 20 years or older from NHANES (2007-2012). The baseline characteristics of participants grouped by lnVc quartiles are presented in **Table 1**. The mean dietary vitamin C intake among these participants was 84.797(2.383) mg/day. For demographic, socioeconomic and dietary condition information, participants in different lnVc quartiles have different Va, Vd, Ve, Zn, Se, gender, race/ethnicity, PIR, annual family income, education level and smoking habits (all *P*<0.05). For thyroid function indices, differences in FT3, TT3, and TT4 were statistically significant (all *P*<0.05).

**Table 1:** Baseline characteristics of the NHANES (2007-2012) study population in lnVc quartiles.

Characreristics	lnVc quartiles					P-value
	Overall	Q1	Q2	Q3	Q4	
N(%)	5878(100)	1472(25.788)	1467(24.916)	1469(24.604)	1470(24.691)	
**lnVc**	[0, 3.037]	[0, 1.508]	(1.508, 1.828]	(1.828, 2.074]	(2.074, 3.037]	
**Vc(mg/d)**	84.797(2.383)**^a^**	17.805(0.333)	47.770(0.432)	89.192(0.526)	187.751(3.193)	<0.0001
**FT4(pmol/L)**	10.116(0.069)	10.160(0.097)	10.129(0.087)	10.168(0.097)	10.005(0.080)	0.214
**FT3(pg/mL)**	3.189(0.011)	3.227(0.021)	3.168(0.017)	3.169(0.016)	3.188(0.016)	0.03
**TSH(uIU/mL)**	2.023(0.074)	1.954(0.087)	2.207(0.234)	1.988(0.050)	1.943(0.082)	0.729
**TT3(nmol/L)**	1.807(0.014)	1.847(0.020)	1.820(0.016)	1.770(0.024)	1.789(0.018)	0.016
**TT4(ug/dL)**	7.788(0.047)	7.900(0.068)	7.824(0.055)	7.785(0.056)	7.638(0.062)	< 0.001
**TGAb(IU/mL)**	7.661(1.625)	4.946(1.491)	9.760(2.990)	10.838(5.288)	5.212(1.240)	0.357
**TPOAb(IU/mL)**	19.442(1.762)	17.122(3.461)	19.045(2.572)	16.739(2.364)	24.959(5.512)	0.591
**FT3/FT4**	1.186(0.008)	1.193(0.011)	1.178(0.011)	1.175(0.012)	1.199(0.011)	0.21
**Age(years)**						0.092
20-40	2112(41.608)**^b^**	561(45.443)	489(38.258)	497(39.040)	565(43.543)	
41-60	1968(38.760)	525(38.304)	500(40.973)	471(38.543)	472(37.219)	
>60	1798(19.632)	386(16.253)	478(20.769)	501(22.417)	433(19.238)	
**Gender**						<0.0001
Female	2861(50.645)	743(52.933)	738(51.301)	769(54.939)	611(43.315)	
Male	3017(49.355)	729(47.067)	729(48.699)	700(45.061)	859(56.685)	
**Race**						0.029
White	2796(69.851)	797(73.619)	702(70.647)	684(70.267)	613(64.696)	
Black	1211(10.687)	290( 9.886)	285( 9.726)	287( 9.918)	349(13.260)	
Mexican	912( 8.317)	181(6.571)	247(8.871)	245(8.428)	239(9.472)	
other race	959(11.145)	204( 9.924)	233(10.755)	253(11.387)	269(12.572)	
**Family income($)**						**< 0.001**
<25,000	1996(25.829)	606(31.851)	488(25.328)	454(22.571)	448(23.292)	
25,000-74,999	2530(42.582)	610(43.467)	654(42.846)	643(44.037)	623(39.944)	
>=75,000	1352(31.588)	256(24.682)	325(31.826)	372(33.392)	399(36.764)	
**Education**						<0.0001
<high school	1590(18.133)	488(23.147)	402(18.479)	355(15.965)	345(14.706)	
High School	1360(23.417)	394(28.443)	356(26.652)	305(19.691)	305(18.614)	
>high school	2928(58.451)	590(48.410)	709(54.869)	809(64.344)	820(66.679)	
**PIR**						< 0.001
<1.0	1216(15.079)	393(18.692)	292(14.134)	265(13.525)	266(13.807)	
1.0-1.9	1598(20.771)	446(25.129)	423(21.531)	372(18.054)	357(18.160)	
2.0-3.9	1545(27.285)	332(26.003)	392(26.771)	418(29.264)	403(27.173)	
>=4.0	1519(36.865)	301(30.177)	360(37.564)	414(39.157)	444(40.859)	
**BMI (kg/m^2^)**						0.137
<25	1670(31.604)	408(29.062)	393(29.131)	414(33.658)	455(34.709)	
25-29.9	2024(33.456)	487(33.661)	523(33.783)	506(32.541)	508(33.825)	
=>30	2184(34.939)	577(37.278)	551(37.086)	549(33.800)	507(31.465)	
**Alcohol (g/d)**						0.178
<=50	5620(94.254)	1405(94.304)	1390(92.138)	1412(95.518)	1413(95.077)	
51-100	211( 4.700)	50(4.915)	63(5.980)	49(3.640)	49(4.241)	
>100	47( 1.046)	17(0.781)	14(1.883)	8(0.842)	8(0.683)	
**Smoke**						<0.0001
never	3113(53.919)	625(43.382)	775(50.188)	824(60.483)	889(62.147)	
former	1525(24.148)	355(23.148)	376(24.728)	418(24.423)	376(24.336)	
now	1240(21.933)	492(33.470)	316(25.084)	227(15.094)	205(13.517)	
**UIC(ug/L)**						0.426
<100	1888(33.542)	486(33.227)	482(35.444)	457(33.030)	463(32.461)	
100-299	2893(48.780)	703(48.474)	710(46.457)	754(51.771)	726(48.462)	
>=300	1097(17.679)	283(18.298)	275(18.099)	258(15.199)	281(19.078)	
**Va(mg/d)**	631.242 (12.921)	422.439 (14.572)	577.928 (14.488)	700.939 (19.612)	833.670 (21.192)	<0.0001
**Vd(mg/d)**	4.694(0.088)	3.542(0.164)	4.603(0.235)	4.998(0.203)	5.688(0.209)	<0.0001
**Ve(mg/d)**	7.916(0.156)	5.621(0.183)	7.223(0.155)	8.815(0.158)	10.115(0.307)	<0.0001
**Se(mg/d)**	112.986(1.216)	101.720(1.596)	112.924(2.466)	113.844(1.713)	123.959(2.136)	<0.0001
**Zn(mg/d)**	12.067(0.188)	10.462(0.405)	11.771(0.282)	12.467(0.199)	13.646(0.334)	<0.0001

**^a^** Weighted mean (SD). **^b^** Actual frequencies (weighted percentages). lnVc, Logarithm-transformed vitamin C; SD, standard deviation; Vc, vitamin C; FT4, free thyroxine; FT3, free triiodothyronine; TSH, thyroid stimulating hormone; TT3, total triiodothyronine; TT4, total thyroxine; TGAb, thyroglobulin antibody; TPOAb, thyroid peroxidase antibody; PIR, poverty to income ratio; BMI, body mass index; UIC, urinary iodine concentration; Va, vitamin A; Vd, vitamin D; Ve, vitamin E; Se, selenium; Zn, zinc.

### 3.2 The correlation between lnVc and thyroid function

The correlation between lnVc and thyroid hormone levels was illustrated in **Table 2**. In Model 1, a negative relationship was observed between lnVc and TT4 [β= -0.208, 95% CI= (-0.321, -0.094), *P*<0.001]. After adjusting for a comprehensive array of factors including age, gender, education level, race/ethnicity, PIR, BMI, UIC, alcohol consumption, annual family income and smoking habits in Model 2, this negative association was remained [β= -0.204, 95% CI= (-0.312, -0.096), *P*<0.001]. Further adjustments for additional confounders in Model 3, including Va, Vd, Ve, Zn and Se, the negative association also remained significant [β= -0.182, 95% CI= (-0.305, -0.059), *P=* 0.006]. Furthermore, when lnVc was categorized, the negative trend remained statistically significant (*P* for trend = 0.001). Compared with individuals in quartile 1(Q1), those in quartile 4(Q4) consistently displayed reduced TT4 levels in all models.

**Table 2:** The associations between lnVc and thyroid function.

	Model 1		Model 2		Model 3	
	β (95%CI)	P	β (95%CI)	P	β (95%CI)	P
TT4 (ug/dL)						
lnVc	-0.208(-0.321,-0.094)	<0.001	-0.204(-0.312,-0.096)	<0.001	-0.182(-0.305,-0.059)	0.006
Categories						
Q1	ref		ref		ref	
Q2	-0.076(-0.217, 0.065)	0.285	-0.059(-0.193, 0.074)	0.368	-0.05(-0.186, 0.085)	0.445
Q3	-0.114(-0.234, 0.006)	0.061	-0.129(-0.253,-0.005)	0.041	-0.116(-0.242, 0.010)	0.070
Q4	-0.261(-0.375,-0.148)	<0.0001	-0.243(-0.361,-0.126)	<0.001	-0.226(-0.348,-0.105)	<0.001
p for trend		<0.0001		<0.001		0.001
TT3 (nmol/L)						
lnVc	-0.057(-0.100,-0.014)	0.011	-0.03(-0.065, 0.005)	0.093	-0.029(-0.074, 0.016)	0.192
Categories						
Q1	ref		ref		ref	
Q2	-0.027(-0.070, 0.016)	0.214	-0.004(-0.041, 0.033)	0.829	-0.005(-0.042, 0.032)	0.779
Q3	-0.077(-0.127,-0.027)	0.003	-0.044(-0.096, 0.009)	0.102	-0.044(-0.102, 0.014)	0.130
Q4	-0.059(-0.108,-0.009)	0.021	-0.034(-0.078, 0.011)	0.128	-0.035(-0.086, 0.016)	0.172
p for trend		0.004		0.053		0.097
FT4 (pmol/L)						
lnVc	-0.045(-0.211,0.120)	0.584	-0.141(-0.292, 0.011)	0.068	-0.11(-0.259, 0.039)	0.141
Categories						
Q1	ref		ref		ref	
Q2	-0.032(-0.226,0.162)	0.744	-0.052(-0.227, 0.123)	0.544	-0.041(-0.221, 0.139)	0.642
Q3	0.008(-0.203,0.219)	0.940	-0.06(-0.261, 0.141)	0.546	-0.048(-0.252, 0.156)	0.628
Q4	-0.155(-0.339,0.029)	0.096	-0.251(-0.429,-0.073)	0.008	-0.234(-0.395,-0.073)	0.007
p for trend		0.153		0.01		0.008
FT3 (pg/mL)						
lnVc	-0.028(-0.070,0.013)	0.171	-0.008(-0.044, 0.027)	0.631	-0.004(-0.041, 0.032)	0.816
Categories						
Q1	ref		ref		ref	
Q2	-0.058(-0.106,-0.011)	0.017	-0.034(-0.075, 0.006)	0.094	-0.033(-0.074, 0.007)	0.099
Q3	-0.058(-0.101,-0.015)	0.010	-0.014(-0.053, 0.024)	0.449	-0.012(-0.054, 0.029)	0.549
Q4	-0.039(-0.089, 0.012)	0.131	-0.026(-0.070, 0.018)	0.238	-0.022(-0.066, 0.021)	0.298
p for trend		0.12		0.356		0.466
TSH (uIU/mL)						
lnVc	-0.06(-0.250,0.129)	0.524	-0.084(-0.259, 0.091)	0.331	-0.036(-0.217, 0.144)	0.680
Categories						
Q1	ref		ref		ref	
Q2	0.253(-0.229,0.736)	0.296	0.232(-0.249, 0.713)	0.330	0.235(-0.250, 0.720)	0.325
Q3	0.034(-0.195,0.263)	0.767	-0.029(-0.260, 0.201)	0.795	0.007(-0.223, 0.237)	0.950
Q4	-0.01(-0.217,0.196)	0.919	-0.02(-0.203, 0.163)	0.825	0.035(-0.175, 0.245)	0.730
p for trend		0.539		0.413		0.721
TPOAb (IU/mL)						
lnVc	4.689(-5.465,14.843)	0.358	6.032( -6.342,18.406)	0.326	8.694( -6.584,23.971)	0.251
Categories						
Q1	ref		ref		ref	
Q2	1.923(-6.597,10.444)	0.652	1.885( -6.587,10.358)	0.651	3.142( -5.761,12.046)	0.470
Q3	-0.383(-9.269, 8.502)	0.931	-0.7( -9.996, 8.596)	0.878	1.324( -9.152,11.800)	0.795
Q4	7.837(-5.614,21.289)	0.247	9.637( -6.304,25.578)	0.225	12.544( -6.267,31.355)	0.180
p for trend		0.302		0.282		0.220
TGAb (IU/mL)						
lnVc	1.519(-4.200,7.237)	0.596	1.443( -5.134, 8.020)	0.656	3.186( -5.904,12.276)	0.475
Categories						
Q1	ref		ref		ref	
Q2	4.813(-1.857,11.483)	0.153	4.719( -2.304,11.743)	0.179	5.14( -2.369,12.650)	0.169
Q3	5.892(-6.206,17.989)	0.332	5.248( -8.046,18.542)	0.424	6.408( -8.377,21.192)	0.377
Q4	0.266(-3.669, 4.201)	0.892	0.47( -4.412, 5.352)	0.844	2.17( -5.368, 9.709)	0.555
p for trend		0.828		0.862		0.611
FT3/FT4						
lnVc	-0.003(-0.024,0.017)	0.746	0.015(-0.005, 0.035)	0.140	0.012(-0.010, 0.033)	0.262
Categories						
Q1	ref		ref		ref	
Q2	-0.015(-0.039,0.010)	0.236	-0.004(-0.028, 0.019)	0.704	-0.006(-0.030, 0.018)	0.607
Q3	-0.017(-0.044,0.009)	0.188	0.006(-0.020, 0.031)	0.639	0.005(-0.022, 0.032)	0.715
Q4	0.007(-0.021,0.034)	0.633	0.022(-0.005, 0.050)	0.110	0.021(-0.007, 0.048)	0.130
p for trend		0.701		0.064		0.077

Model 1 was adjusted no covariates;.

Model 2 was adjusted for age, gender, education level, race/ethnicity, PIR, BMI, UIC, alcohol consumption, annual family income, smoking habits.

Model 3 was further adjusted for Va, Vd, Ve, Zn and Se.

CI, confidence interval; Ref, reference.

In Model 1, lnVc exhibited a negative correlation solely with TT3 [β= -0.057, 95% CI=(-0.100, -0.014), *P*=0.011]. When lnVc was treated as a categorical variable in Model 1, this inverse relationship remained significant (*P* for trend = 0.004). However, upon adjusting for potential confounders in Models 2 and 3, this association was no longer observed.

In contrast, when lnVc was used as a categorical variable, the participants in quartile 4(Q4) had a lower FT4 than those in quartile 1(Q1) in Model 2 (*P* for trend = 0.01) and Model 3 (*P* for trend = 0.008).

### 3.3 RCS analysis

**Figure 2:**
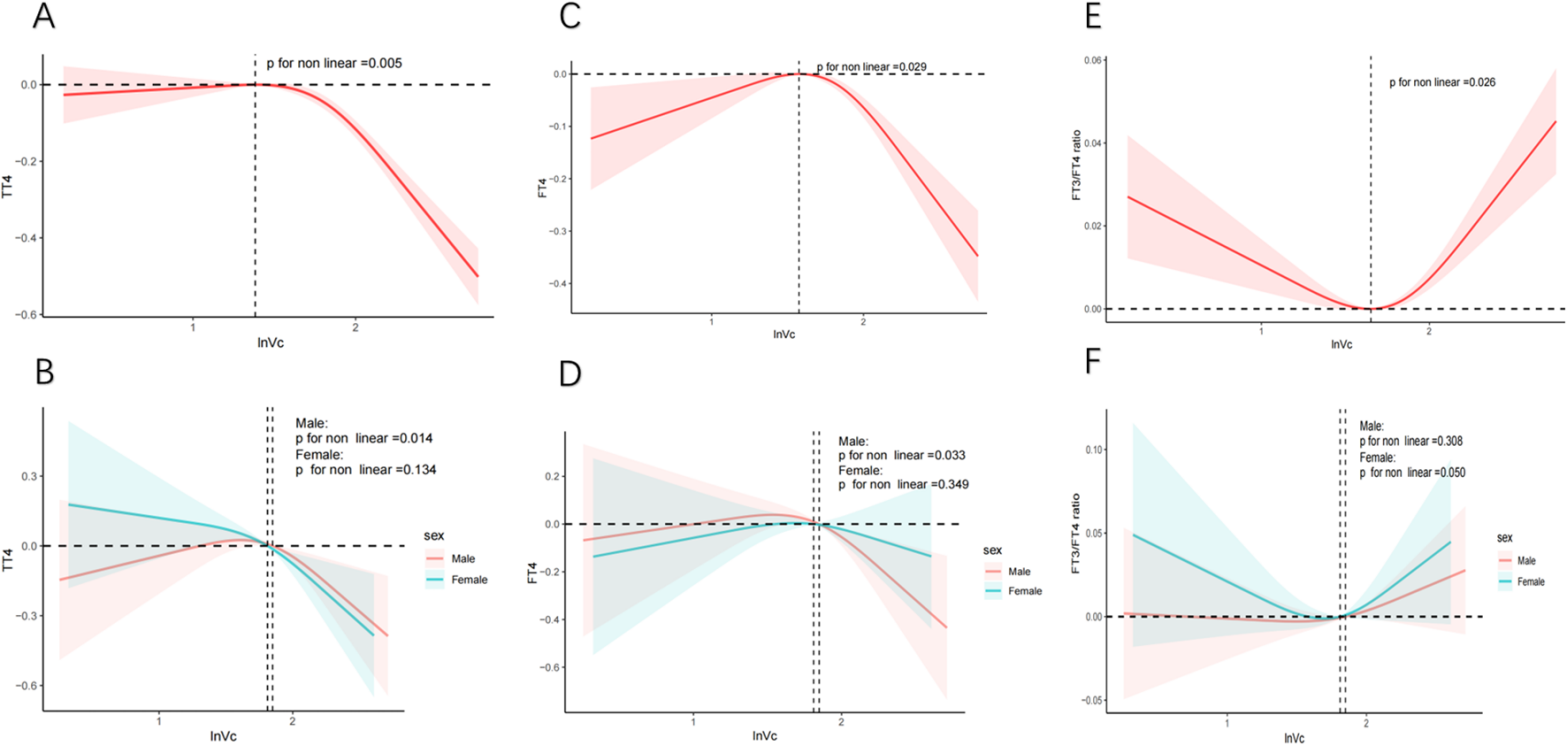
RCS analysis of nonlinear association between lnVc and thyroid function. Notes: The solid line represents the RCS curves between lnVc and thyroid function. The red or blue shaded area symbolize the 95% confidence intervals. Models adjust for age, gender, education level, race/ethnicity, PIR, BMI, UIC, alcohol consumption, annual family income, smoking habits,Va, Vd, Ve, Zn and Se. **(A)** TT4 of total adults; **(B)** TT4 of male or female adults; **(C)** FT4 of total adults; **(D)** FT4 of male or female adults; **(E)** FT3/FT4 of total adults; **(F)** FT3/FT4 of male or female adults.

The relationship between lnVc and thyroid hormone levels was further scrutinized through the application of RCS curves. Initially, upon adjusting for potential confounding factors, we observed a reverse U-shaped curve association (P for nonlinear = 0.029) between lnVc and FT4 (**Figure 2C**). Regarding the strong inverted U-shaped relationship, **Figure 2C** showed an increase of FT4 levels with increasing lnVc levels, which reached the highest around 1.575 and then decreased thereafter. In addition, the inflection point for lnVc levels in females was 1.811 (*P* for nonlinear = 0.033) (**Figure 2D**).

Second, an U-shaped curve relationship was noticed (*P* for nonlinear = 0.026) between lnVc and FT3/FT4 ratio (**Figure 2E**). When the lnVc level was 1.652, the FT3/FT4 ratio was the lowest. And, there was no significant difference between the genders (**Figure 2F**).

The correlation between lnVc and TT4 exhibited a distinct reversed L-shaped curve pattern in total participants(*P* for nonlinear = 0.005, **Figure 2A**) and male adults (*P* for nonlinear = 0.014, **Figure 2B**). However, we did not find a nonlinear correlation among TSH, FT3, TT3, TPOAb, TgAb, and lnVc (**Supplementary Figures S2**).

### 3.4 Subgroup analysis

**Figure 3:**
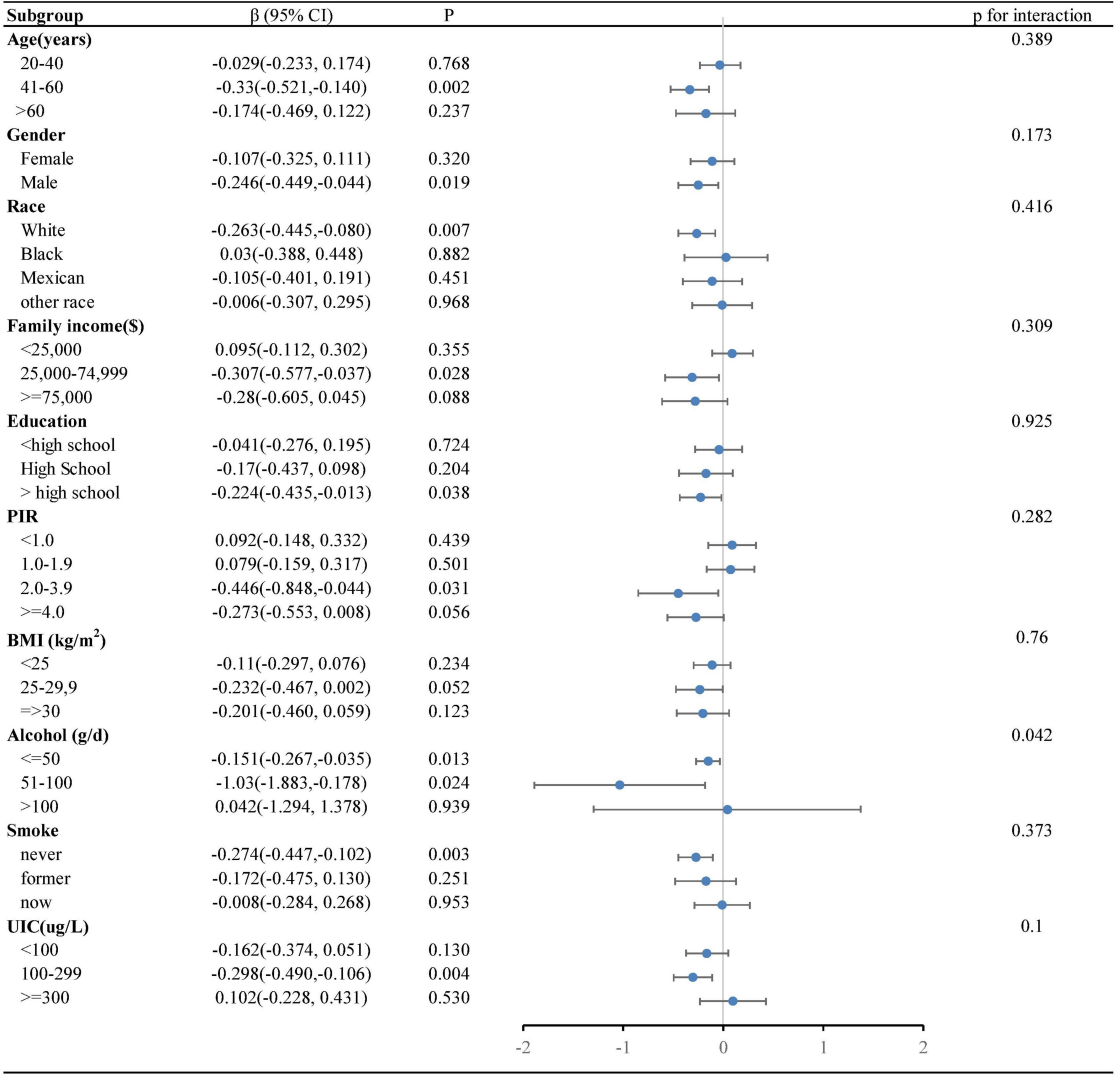
Forest plot of the relationship between lnVc and TT4 in each subgroup. Each subgroup adjusted for all factors (age, gender, education level, race/ethnicity, PIR, BMI, UIC, alcohol consumption, annual family income, smoking habits,Va, Vd, Ve, Zn and Se) except the stratification factor itself.

Our research revealed a negative correlation between lnVc and TT4. To further explore this correlation among diverse population groups, we conducted a subgroup analysis utilizing multivariable linear regression. The outcomes of this stratified analysis were presented in **Figure 3** and **Table 3**.

**Table 3:** Relationship between lnVc and TT4 in each subgroup.

Characreristics	lnVc quartiles					
	Q1	Q2	Q3	Q4	p for trend	p for interaction
**Age(years)**						0.291
20-40	ref	0.176(-0.066, 0.418)	0.066(-0.211, 0.343)	-0.074(-0.303, 0.156)	0.395	
41-60	ref	-0.216(-0.439, 0.008)	-0.285(-0.557,-0.014)	-0.373(-0.631,-0.115)	0.016	
>60	ref	-0.171(-0.445, 0.103)	-0.188(-0.451, 0.075)	-0.197(-0.515, 0.120)	0.224	
**Gender**						0.347
Female	ref	0.045(-0.165, 0.255)	-0.117(-0.329, 0.096)	-0.165(-0.373, 0.042)	0.068	
Male	ref	-0.138(-0.354, 0.079)	-0.112(-0.304, 0.079)	-0.273(-0.513,-0.033)	0.045	
**Race**						0.124
White	ref	-0.101(-0.274, 0.072)	-0.196(-0.366,-0.027)	-0.354(-0.525,-0.183)	<0.001	
Black	ref	-0.246(-0.594, 0.103)	0.097(-0.299, 0.492)	0.003(-0.437, 0.444)	0.616	
Mexican	ref	0.116(-0.255, 0.488)	0.06(-0.267, 0.387)	0.033(-0.328, 0.393)	0.983	
other race	ref	0.306(-0.225, 0.836)	0.055(-0.306, 0.416)	0.095(-0.238, 0.428)	0.998	
**Family income($)**						0.782
<25,000	ref	0.039(-0.265, 0.344)	0.015(-0.300, 0.331)	0.01(-0.297, 0.318)	0.97	
25,000-74,999	ref	-0.109(-0.362, 0.145)	-0.095(-0.366, 0.176)	-0.32(-0.608,-0.031)	0.055	
>=75,000	ref	-0.035(-0.436, 0.365)	-0.25(-0.600, 0.100)	-0.295(-0.682, 0.093)	0.047	
**Education**						0.665
<high school	ref	0.006(-0.288, 0.300)	0.144(-0.189, 0.477)	-0.087(-0.394, 0.220)	0.91	
High School	ref	-0.129(-0.466, 0.207)	-0.034(-0.280, 0.212)	-0.217(-0.541, 0.107)	0.254	
> high school	ref	-0.047(-0.239, 0.145)	-0.205(-0.421, 0.011)	-0.26(-0.473,-0.048)	0.01	
**PIR**						0.944
<1.0	ref	0.06(-0.375, 0.494)	0.061(-0.403, 0.524)	0.082(-0.319, 0.482)	0.713	
1.0-1.9	ref	0.087(-0.194, 0.368)	0.086(-0.212, 0.384)	-0.075(-0.333, 0.184)	0.7	
2.0-3.9	ref	-0.226(-0.547, 0.096)	-0.263(-0.630, 0.104)	-0.332(-0.809, 0.146)	0.172	
>=4.0	ref	-0.009(-0.335, 0.318)	-0.185(-0.483, 0.112)	-0.321(-0.664, 0.022)	0.022	
**BMI (kg/m^2^)**						0.692
<25	ref	0.069(-0.201, 0.338)	0.014(-0.185, 0.213)	-0.159(-0.378, 0.060)	0.11	
25-29,9	ref	-0.182(-0.499, 0.135)	-0.261(-0.554, 0.031)	-0.238(-0.515, 0.039)	0.075	
=>30	ref	-0.068(-0.335, 0.198)	-0.103(-0.354, 0.148)	-0.292(-0.581,-0.003)	0.056	
**Alcohol (g/d)**						0.004
<=50	ref	0.018(-0.126, 0.162)	-0.093(-0.214, 0.028)	-0.186(-0.304,-0.068)	0.002	
51-100	ref	-1.117(-2.038,-0.195)	-0.788(-1.887, 0.312)	-1.011(-2.206, 0.184)	0.173	
>100	ref	-0.775(-1.922, 0.371)	0.772(-0.431, 1.975)	-0.135(-1.783, 1.512)	0.908	
**Smoke**						0.937
never	ref	-0.084(-0.293, 0.125)	-0.163(-0.384, 0.058)	-0.276(-0.484,-0.069)	0.012	
former	ref	-0.014(-0.308, 0.281)	-0.17(-0.444, 0.104)	-0.168(-0.513, 0.178)	0.212	
now	ref	-0.011(-0.296, 0.274)	0.058(-0.283, 0.399)	-0.151(-0.518, 0.216)	0.599	
**UIC**						0.734
<100	ref	-0.048(-0.354, 0.258)	-0.1(-0.324, 0.123)	-0.223(-0.519, 0.073)	0.122	
100-299	ref	-0.132(-0.346, 0.083)	-0.188(-0.409, 0.033)	-0.312(-0.534,-0.090)	0.009	
>=300	ref	0.129(-0.261, 0.519)	0.068(-0.318, 0.455)	0.016(-0.343, 0.376)	0.971	

Data are presented as β (95% CI). Each subgroup adjusted for all factors (age, gender, education level, race/ethnicity, PIR, BMI, UIC, alcohol consumption, annual family income, smoking habits,Va, Vd, Ve, Zn and Se) except the stratification factor itself. CI, confidence interval; Ref, reference.

In the subgroup analysis (**Figure 3**), alcohol consumption plays a pivotal role in modulating the association between lnVc and TT4. Remarkably, this significant association was more apparent in subjects with lower alcohol consumption[β= -0.151, 95% CI= (-0.267, -0.035), *P=*0.013, *P* for interaction = 0.042]. Furthermore, when lnVc was used as a categorical variable (**Table 3**), the groups with lower alcohol consumption (< 50 g/d) and higher lnVc consumption showed a more substantial reduction in TT4 levels compared to other groups[β= -0.186, 95% CI= (-0.304, -0.068), *P =* 0.002, *P* for interaction = 0.004].

## 4 Discussion

This cross-sectional study was conducted to investigate the potential association between vitamin C intake and thyroid function among 5,878 participants from the NHANES between 2007 and 2012. The findings of this study demonstrated a significant inverse correlation between vitamin C intake and TT4 levels. Furthermore, the subgroup analysis and interaction terms suggested that the association was more pronounced in subjects with lower alcohol consumption. In addition, our investigation also uncovered a nonlinear relationship between vitamin C and thyroid function markers, including TT4, FT4,and the FT3/FT4 ratio.

In previous studies, most scholars focused on studying the relationship between vitamin C intake and thyroid cancers (16, 26), goitre (17), benign thyroid diseases (27), hyperthyroidism and hypothyroidism (28), with few exploring the effects of vitamin C intake on thyroid function in normal individuals. Karimi et al. (29) showed that the concentration of anti-thyroid peroxidase antibodies significantly decreased in autoimmune thyroiditis patients treated with vitamin C compared to those receiving a placebo. Jubiz et al. (30) evaluated the effect of vitamin C administration in hypothyroid patients with elevated TSH levels, showing that vitamin C can increase serum T4, T3, and decrease TSH. The findings of these studies support the potential role of vitamin C in the treatment of thyroid-related diseases.

Our research revealed a significant inverse correlation between vitamin C intake and TT4, but we did not observe significant differences in TPOAb and TSH levels between the four groups. This could be attributed to several factors. First, it is possible that the participants in our study did not have sufficient vitamin C intake. Low levels of vitamin C in the body may limit its beneficial effects on thyroid function, even with supplementation. Furthermore, the dose and duration of vitamin C intake in our study may have been insufficient to observe significant changes in TPOAb and TSH levels. In the studies mentioned, participants were administered 500mg of vitamin C daily for months. In contrast, our study only used the average vitamin C intake over a period of two days. Longer-term and higher-dose vitamin C supplementation may be necessary to observe significant improvements in thyroid function and auto-antibody levels. Moreover, our study population consisted of individuals with normal thyroid function. It is possible that the effects of vitamin C on thyroid function may be more pronounced in individuals with pre-existing thyroid conditions.

In our study, we found an inverted U-shaped curve relationship between vitamin C and FT4, while an U-shaped curve relationship was noticed between vitamin C and FT3/FT4 ratio. An increased of FT4 levels with increasing lnVc levels, which reached the highest around 1.575 and then decreased thereafter. When the lnVc levels were less than 1.652, the FT3/FT4 ratio decreased with the increase of lnVc levels. The Food and Drug Administration (FDA) increased the vitamin C recommendations to 90 mg/day for men and 75 mg/day for women. And the study suggests that raising the existing daily recommended amount of vitamin C to 200 milligrams per day would have a positive impact on the function of the immune system (31). Excessive intake of vitamin C may also have adverse side effects, such as kidney stones, nausea, and diarrhea (32, 33).

The subgroup analysis and interaction terms indicated that the association between vitamin C intake and TT4 levels was more apparent in subjects with lower alcohol consumption. This finding suggests that alcohol consumption plays a pivotal role in modulating the association between vitamin C and thyroid function. Previous studies have shown that vitamin C levels are decreased in alcoholics, excessive alcohol consumption can interfere with the absorption and utilization of vitamin C, leading to its depletion in the body (34–37).

Moreover, gender-specific subgroup analysis showed that women are more likely to be affected from vitamin C intake in maintaining healthy thyroid function. However, no significant interaction effects were observed. Previous studies have demonstrated that the prevalence of thyroid disorders, including hypothyroidism, hyperthyroidism (38), thyroid nodules (39) and thyroid cancer (40), are vary significantly between men and women. These finding could be attributed to gender-related differences in thyroid physiology and metabolism, as well as dietary habits and hormonal status (41–43). Metsios et al. (44) found that a brief exposure to moderate passive smoke significantly increased the TT3 and FT4 levels compared to the control group. Gruppen et. al (45) studies also demonstrated that smokers with ≤20 cigarettes per day were associated with higher serum FT4, FT3 and lower TSH compared to nonsmokers. In our study, subgroup analysis revealed that the impact of vitamin C on thyroid function was particularly evident among non-smokers. This suggests that smokers may benefit more from increasing their intake of vitamin C to maintain healthy thyroid function.

However, there are also limitations to using the NHANES database for this research. Firstly, the vitamin C intake relies on self-reported dietary intake data, which may be subject to inaccuracies and biases. This can affect the reliability and validity of the findings. Secondly, even though we have tried our best to eliminate potential confounders, there are still some factors that cannot be fully controlled. These factors may have an impact on the relationship between vitamin C and thyroid function. Finally, we are unable to firmly establish the precise causal relationship between vitamin C intake and thyroid function due to the retrospective cross-sectional nature of this study.

## 5 Conclusion

In conclusion, this cross-sectional study suggested the association between vitamin C and thyroid function. A high level of vitamin C intake is associated with a decrease in TT4 levels among adults in the United States, and the association was more pronounced in subjects with lower alcohol consumption. In addition, our analysis revealed a nonlinear correlation between the intake of vitamin C and the levels of TT4, FT4, and FT3/FT4 ratio. In the future, more studies are needed to further explore the causal relationship.

## Data Availability

The original contributions presented in the study are included in the article/**Supplementary Material**, further inquiries can be directed to the corresponding author/s.
