# Supplementary figures and images for: Association between vitamin C intake and thyroid function among U.S. adults: a population-based study
Source: Front Endocrinol (Lausanne). 2024 Nov 7;15:1462251. doi: 10.3389/fendo.2024.1462251 (PMC11578698; doi:10.3389/fendo.2024.1462251)

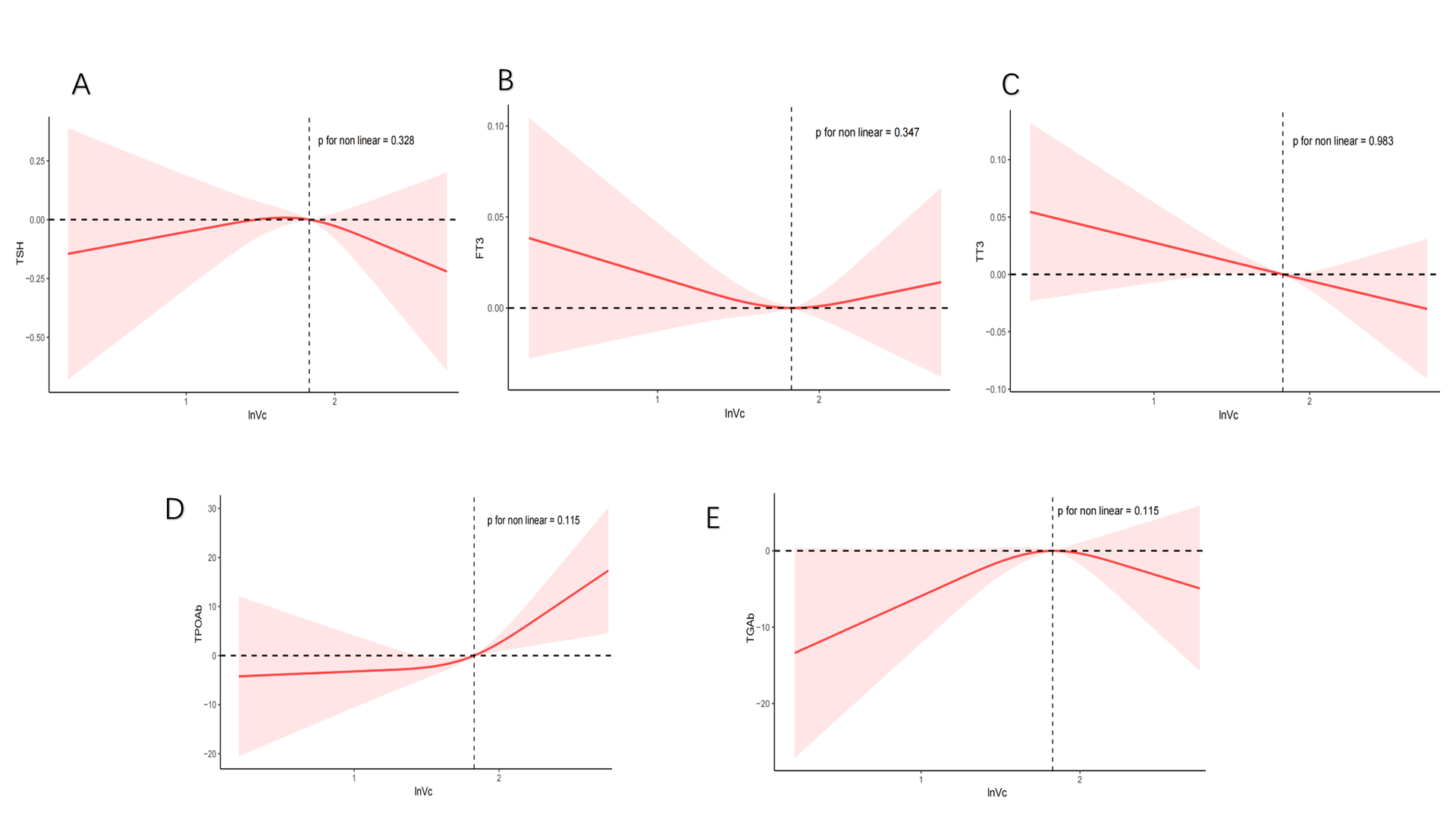

Supplement: Supplementary file 1 [file Image1.tiff]
